# Trigeminal Neuralgia Is a Dementia Risk Factor: A Retrospective Cohort Study

**DOI:** 10.3390/ijerph19106073

**Published:** 2022-05-17

**Authors:** Yung-Han Cheng, Chieh-Hsin Wu, Wei-Ting Wang, Ying-Yi Lu, Ming-Kung Wu

**Affiliations:** 1 Division of Neurosurgery, Department of Surgery, Kaohsiung Medical University Hospital, Kaohsiung 807, Taiwan; destiny310087@livemail.tw (Y.-H.C.); wujoeys@gmail.com (C.-H.W.); 2 Department of Surgery, School of Medicine, College of Medicine, Kaohsiung Medical University, Kaohsiung 807, Taiwan; 3 Department of Radiology, Tri-Service General Hospital, National Defense Medical Center, Taipei City 11490, Taiwan; a8915g@gmail.com; 4 Department of Dermatology, Kaohsiung Veterans General Hospital, Kaohsiung 813, Taiwan; actinp@hotmail.com; 5 Department of Nursing, Shu-Zen Junior College of Medicine and Management, Kaohsiung 821, Taiwan; 6 Department of Psychiatry, Kaohsiung Chang Gung Memorial Hospital and Chang Gung University College of Medicine, Kaohsiung 833, Taiwan; 7 Department of Health and Beauty, Shu-Zen Junior College of Medicine and Management, Kaohsiung 821, Taiwan

**Keywords:** dementia, trigeminal neuralgia, risk

## Abstract

*Background*: Dementia, a worldwide public-health issue, is regarded as a disorder rather than a normal aging process. Trigeminal neuralgia (TN) is a chronic debilitating pain disorder that impairs daily activities. Both are most prevalent in females and in patients older than 50 years. Recent studies reveal that pain and dementia may have a reciprocal interaction with each other. *Objective*: In response, we estimated whether adults with TN have an increased dementia risk. *Methodology*: By means of Taiwan’s National Health Insurance Research Database, between 1996 and 2010, 762 patients aged over 50 years in the TN group were matched with 3048 patients in the non-TN group at a ratio of 1:4. Kaplan–Meier method and Cox proportional hazard regression models were also used to determine the cumulative incidence and compare the hazard ratios of dementia in each group. *Results*: The incidence of dementia was higher in the TN group compared to the non-TN group. After adjusting for covariates, the TN group had a 4.47-fold higher risk of dementia compared to the non-TN group. Additionally, the impact of TN on dementia risk was larger in young-aged patients than in old-aged patients. As well, the age at the time of dementia diagnosis was younger in the TN group compared to the non-TN group. *Conclusions*: TN is a dementia risk factor. Given the lack of a curative therapy for dementia, early identification of TN patients may help to prevent dementia sequelae.

## 1. Introduction

Dementia is a worldwide health issue. The global incidence of cognitive impairment and memory loss after people move to their 4th decade of life is currently increasing. The US has a 15% prevalence of dementia in people older than 68 years [1]. In Taiwan, the dementia population exceeded 270,000 at the end of 2017, and the dementia population is expected to increase threefold within the next 50 years. Although its prevalence increases with age, dementia is considered a disorder rather than a normal aging process [2]. Additionally, since dementia is an irreversible, progressive disorder characterized by the acquired loss of cognitive abilities, it can significantly impair social and physical functions [3]. The two most common dementia types, Alzheimer’s disease and vascular dementia, have similar pathophysiology and often occur simultaneously. Dementia is a leading cause of disability and dependency in the elderly, and the physical, emotional, and economic burdens in this age group are particularly high. Given the current lack of an effective curative therapy for dementia, early identification of high-risk patients may be the most effective approach to preventing dementia sequelae [4].

Trigeminal neuralgia (TN), a so-called *tic douloureux*, is a chronic debilitating pain disorder characterized by unilateral paroxysmal, stabbing, stimulus-evoked orofacial pain. The effects of TN are often limited to one or more branches of the trigeminal nerve [5], and classic TN usually results from neurovascular compression of primary sensory afferents in the root entry zone [6,7]. However, TN may be secondary to a space-occupying lesion, multiple sclerosis, maxillary sinusitis, or periodontitis [8,9,10]. Severe TN can impair daily activities and can cause depressive disorder or other comorbidities [11]. The incidence of TN is similar to that of dementia, and both are most prevalent in females and in patients older than 50 years [12,13].

Recent studies reveal that pain and dementia may have a reciprocal interaction with each other [3]. A recent study hypothesized that TN may be, in addition to memory loss, a presentation of temporal lobe epilepsy [14]. Almost half of dementia patients complain of pain sensations, whereas TN also co-occurs with cognitive dysfunction in brainstem lesions [15]. Although growing evidence suggests that chronic pain increases the risks of cognitive decline and dementia, no studies have investigated the relationship between TN and dementia. Therefore, the aim of this study was to use the Taiwan National Health Insurance Research Database (NHIRD) to investigate whether adults with TN have an increased dementia risk.

## 2. Materials and Methods

### 2.1. Data Sources

The Taiwan National Health Insurance (NHI) program was implemented in March 1995 and currently covers more than 99% of the 23.74 million residents of Taiwan. The National Health Research Institute publishes the NHIRD for use in medical research. The NHIRD is an encrypted secondary database containing all NHI records. Since the NHIRD contains records for a large sample of patients, it is considered representative of real-world data. Therefore, this study used the Longitudinal Health Insurance Database 2010 (LHID2010), which contains data for a sample of 1,000,000 beneficiaries randomly selected from the original NHIRD. All diseases were coded based on the International Classification of Diseases, Ninth Revision, Clinical Modification (ICD-9-CM).

### 2.2. Ethics Statement

The collection of reimbursement claims data in this study was limited to the Taiwan NHIRD. The study was assessed and approved by the Institutional Review Board (IRB) of Chang Gung Medical Foundation (IRB No. 201901117B0) according to the Declaration of Helsinki. The requirement of informed consent was waived in accordance with IRB regulations.

### 2.3. Study Population

**Figure 1:**
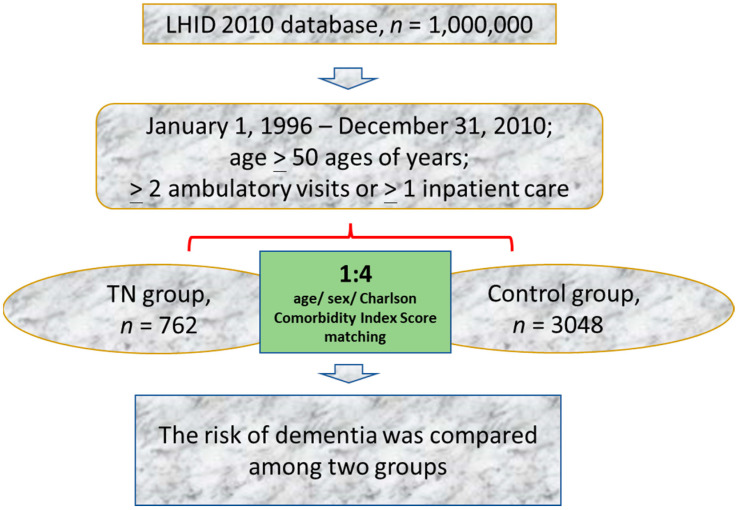
Flowchart of the study procedure. LHID, Longitudinal Health Insurance Database.

During 1996–2010, this study recruited 762 patients with TN aged more than 50 years. To increase the consistency of characteristics in the study population, TN was defined as a record of an ICD-9-CM code 350.1 assigned by a neurologist in two or more consecutive ambulatory visits or in one or more hospital inpatient stays. To minimize the possibility of including TN other than classic TN, patients were excluded if they had any history of benign or malignant neoplasm of the nervous system (ICD-9-CM code 191, 192, and 225) or multiple sclerosis (ICD-9-CM code 340) [16,17]. The index date was the date of the first TN diagnosis. Figure 1 shows the results of the propensity score matching procedure used to match the 762 patients in the TN group with 3048 patients in the control group without TN at a ratio of 1:4.

### 2.4. Main Outcome

The TN and non-TN groups were followed up until the date of the first diagnosis of dementia or until 31 December 2010. A diagnosis of dementia was defined as a record of ICD-9-CM codes 290, 294.1, 331.0, and 331.2 entered by a neurologist [18].

### 2.5. Comorbidities

Comorbidities identified as potential confounders in this study included hyperlipidemia (ICD-9-CM code 272), hypertension (ICD-9-CM codes 401–405), heart failure (ICD-9-CM code 428), coronary artery disease (ICD-9-CM code 410–414), chronic obstructive pulmonary disease (ICD-9-CM code 491, 492, 494 and 496), traumatic brain injury (ICD-9-CM code 800, 803, 804 and 850–854), depression (ICD-9-CM code 296.2, 296.3, 300.4 and 311), Parkinson’s disease (ICD-9-CM code 332) and stroke (ICD-9-CM code 430–438). The Charlson comorbidity index (CCI) was used to classify the severity of comorbidity into four levels: 0, 1–2, 3–4, and ≥5. Medications (anti-convulsant, anti-depressant, or baclofen) prescribed for TN treatment were also analyzed.

### 2.6. Statistical Analysis

Chi-square test and Student t-test were used to compare categorical and continuous variables of demographic characteristics between the TN and non-TN groups, as appropriate. In each group, overall incidence rates specific to age and sex were estimated per 1000 person-years. Kaplan–Meier method was also used to determine the cumulative incidence of dementia in each group, and the proportions were calculated by log-rank test. Cox proportional hazard regression models were used to compare hazard ratios (HRs) and 95% confidence intervals (CIs) for dementia between the two groups after adjustment for age, sex, Charlson comorbidity index, and relevant comorbidities (hyperlipidemia, hypertension, heart failure, coronary artery disease, chronic obstructive pulmonary disease, traumatic brain injury, depression, Parkinson’s disease and stroke), and medications in the multivariable model. A *p*-value < 0.05 was considered statistically significant. Statistical Analysis Software 9.4 (SAS Institute, Cary, NC, USA) was used to process all data analyses.

## 3. Results

### 3.1. Baseline Demographic Characteristics of Patients with and without TN

The 3810 patients enrolled in this study included 762 patients in the TN group and 3048 patients matched by age, sex, and Charlson Comorbidity Index Score in the non-TN (control) group. Table 1 shows that most (62.99%) patients were female. Compared to the non-TN group, the TN group had a higher prevalence of comorbidities (hyperlipidemia, hypertension, heart failure, coronary artery disease, chronic obstructive pulmonary disease, traumatic brain injury, depression, Parkinson’s disease, and stroke). The TN group also had significantly higher use of anti-convulsants (85.70 vs. 18.96 in the non-TN group, *p* < 0.001), anti-depressants (20.34 vs. 6.23 in the non-TN group, *p* < 0.001), baclofen (69.03 vs. 37.30 in the non-TN group, *p* < 0.001).

### 3.2. Incidence and Risk of Dementia after TN

The incidence of dementia was significantly (*p* < 0.001) higher in the TN group (11.02%, 84 patients) compared to the non-TN group (6.4%, 195 patients) (Table 2). Additionally, the time from index date to dementia diagnosis was significantly shorter in the TN group (3.7 years) compared to the non-TN group (11.4 years). Finally, age at the time of dementia diagnosis was significantly younger in the TN group compared to the non-TN group (72.2 vs. 76.3 years, respectively; *p* < 0.001).

Table 3 shows that, after adjusting for age, sex, CCI score, comorbidities, and medications, the TN group had a 4.47-fold higher risk of dementia compared to the non-TN group (19.16 vs. 4.35 per 1000 person-years, respectively) during the follow-up period. Stratified analyses also revealed a higher dementia risk in the TN group. In TN cohorts, sex-specific analyses revealed that females had a higher incidence of dementia per 1000 person-years (19.45 vs. 18.65 in males) and a similar risk of dementia (adjusted HR = 4.78, 95% CI: 3.00–7.61 versus adjusted HR = 4.24, 95% CI: 2.29–7.83 in males, *p* < 0.001). Age-specific analysis consistently showed that the incidence of dementia substantially increased with age. However, the impact of TN on dementia risk was larger in young-aged patients (adjusted HR = 6.07, 95% CI = 2.52–14.62) than in old-aged patients (adjusted HR = 4.57, 95% CI = 3.04–6.88).

**Figure 2:**
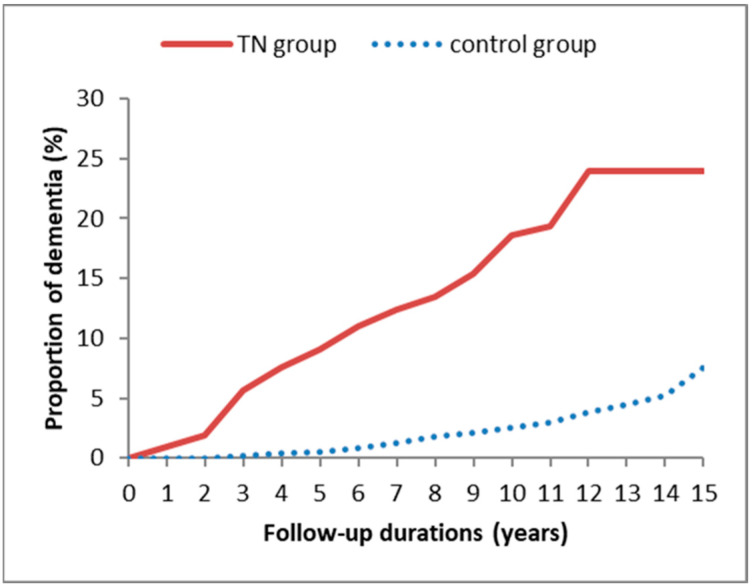
Kaplan–Meier curves for dementia risk in patients with and without trigeminal neuralgia during a 15-year follow-up period.

In Figure 2, Kaplan–Meier method with the log-rank test further revealed that the TN group had a higher cumulative incidence rate of dementia compared to the non-TN control group (*p* < 0.001).

### 3.3. Risk Factors for Dementia in TN Group

Since TN patients tended to develop dementia compared to the controls, we further tried to identify the characteristics of TN patients who easily developed dementia. Several predictors of dementia are presented in Supplementary Table S1. In TN groups, dementia risk increased with age, Charlson comorbidity index score, and with the presence of depression, Parkinson’s disease, traumatic brain injury, and coronary artery disease. The effect of TN and comorbidities on dementia risk are shown in Supplementary Table S2, which demonstrates that these comorbidities jointly contributed to the increased dementia risk in the TN group.

## 4. Discussion

This study is the first large retrospective cohort study to demonstrate an association between TN and dementia: 11.2% of TN patients older than 50 years had dementia. Compared to the non-TN control group, the TN group had a 4.47-fold higher dementia risk after adjustment for comorbidities and medications. The impact of TN on dementia risk was larger in young-aged patients than in old-aged patients. Additionally, in the TN group, dementia was more common in females than in males and was more common in older patients than in younger patients. Notably, Parkinson’s disease, traumatic brain injury, and coronary artery disease had synergistic effects on dementia incidence in TN patients.

Our data showed that the impact of TN on dementia risk was largest in the young-aged cohort (aged 50–59 years) (Table 3). However, the incidence rate of dementia was still higher in the old-aged cohort of TN patients. Compared to TN patients in the young-aged cohort, those in the old-age cohort tended to have more comorbidities that contributed to dementia risk. That is, the impact of TN on cognition loss may not be significantly associated with advanced age. Although there was no previous study revealed the impact of age on the incidence of dementia in TN patients, several studies suggested that dementia is an age-dependent disease and increases in an exponential manner [19]. Other than high blood pressure during midlife, high systolic pressure and low diastolic pressure in older age contribute to late-life dementia [20]. The age-dependent cellular changes result in the impairment of neuronal identity [21]. Therefore, the comparison results in our study suggest that the pathophysiology of dementia may differ between younger and older TN patients.

Although no distinct pathways of the increased dementia risk observed in the TN patients in this study were identified, several possible mechanisms exist. First, TN is a notorious chronic pain disorder that often impairs health and activities of daily living [22]. Chronic pain is an important contributor to cognitive impairment [23,24]. Chronic pain in older adults is the most common cause of impaired activities of daily living and poor sleep quality. Thus, chronic pain can cause psychiatric problems (e.g., depressive disorder and anxiety disorder), sleep disturbance, physical/social dysfunction, and can even cause cognitive impairment [25]. Elizabeth et al. reported that, in dementia patients admitted to acute care, pain symptoms are often underestimated and/or undertreated and are often psychiatric manifestations of dementia [26]. Rajkumar et al. also reported that, by reducing pain severity, regular treatment with analgesics improved health and quality of life in dementia patients [27]. Several studies have reported that chronic pain increases the risks of cognitive decline and dementia [23,24]. Chronic pain may cause neural degeneration or loss in the cortex or hippocampus by increasing cortisol levels, which then leads to memory dysfunction [28,29,30]. Brain magnetic resonance imaging studies have shown that a prolonged duration of pain can decrease the volume of brain gray matter [31]. Therefore, TN patients are prone to cognitive decline and dementia.

Second, chronic inflammation contributes to TN formation [32]. The release of inflammatory neuropeptides and excitation of the trigeminal vascular system can induce local trigeminal neuropathy [33]. Inflammation has a critical role in the interplay between TN and several diseases, including maxillary sinusitis, periodontitis, periostitis, and dental cysts [8]. Alzheimer’s disease is a well-known progressive neuroinflammatory disease that impairs cognitive function and memory [34]. Proinflammatory cytokine activation causes brain dysfunction not only by triggering the release of oxidative stress and neurotoxic derivatives but also by increasing microglial activation and neuronal apoptosis [35,36]. Thus, TN could increase dementia risk via chronic inflammation. Third, depression increased dementia risk in the TN patients in this study. Wu et al. demonstrated that TN increased the risks of depressive disorder, anxiety disorders, and sleep disturbance [37,38]. Notably, the incidence of depression, anxiety, and functional disabilities increased with the severity of pain [39]. Chronic depression is a well-known predisposing factor in dementia [40]. The synthesis of the NMDA glutamate agonist, quinolinic acid, and kynurenine metabolites induces oxidative stress and chronic inflammation status [35]. Additionally, chronic stress can suppress the adiponectin-notch pathway, which is essential for hippocampal neurogenesis and cognitive function. Chronic inflammation and the adiponectin-notch pathway are both involved in the pathogenesis of cognitive dysfunction associated with depression [41]. Therefore, TN may increase dementia risk by contributing to depression disorder.

Additionally, concomitant diseases can jointly influence the incidence of dementia [42,43,44] (Supplementary Tables S1 and S2). Several comorbidities are known to increase the risk of dementia. For example, the long-term effects of multiple vascular risk factors can thicken vessel walls and cause endothelial dysfunction, which then induces oxidative stress and tau-protein hyperphosphorylation [45]. Parkinson’s disease induces not only the formation of cortical Lewy bodies but also the formation of amyloid-β plaques [46]. Accumulation of amyloid-β plaques and amyloid angiopathy can cause cognitive impairment, which then results in dementia [47]. Traumatic brain injury also induces tau-protein pathological changes and deposition of amyloid-β plaques [48,49,50]. In the study, the dementia incidence increased with the presence of Parkinson’s disease, traumatic brain injury, and coronary artery disease in TN patients.

This study is the first population-based study with a large sample size to investigate the interplay between TN and dementia risk. A noted limitation is that diagnoses of TN and dementia were identified by ICD-9-CM codes in claims records. Hence, the actual number of patients with both TN and dementia may have been higher since patients with untreated TN may also have undiagnosed dementia. This problem would occur in the use of any electronic health insurance database in any country. Another limitation is that since the dataset in this study did not include pain severity and dementia score, investigating the association between TN severity and outcome was difficult. In addition, the diagnosed code for diseases was updated from ICD-9-CM to ICD-10-CM in 2016 in Taiwan. To prevent the influence of code change on the analysis, we only used LHID 2010 to investigate the association between trigeminal neuralgia and the risk of dementia. However, the databases had already been used for scientific research in various other studies of dementia; therefore, our study adds additional scientific information [37,51]. A final limitation is that the database lacked detailed data for dementia risk factors, including education level, relevant genetic factors, family history, diet and exercise habits, tobacco and alcohol use, and body mass index, which may have influenced the present results [52].

## 5. Conclusions

This work is the first cohort study to demonstrate that dementia risk is increased in TN patients, especially in TN patients with depression, Parkinson’s disease, traumatic brain injury, and coronary artery disease. The role of TN in dementia risk was larger in young-aged (50–59 years) patients than in old-aged patients. Since neuronal loss and brain atrophy always occur before clinical symptoms of dementia appear, early evaluation and prompt intervention for TN are advised to minimize subsequent impairment of cognitive function or development of dementia.

## Figures and Tables

**Table 1:** Comparison of demographic characteristics between trigeminal neuralgia (TN) patients and non-TN patients.

Characteristics	With Trigeminal Neuralgia (n = 762)	Without Trigeminal Neuralgia (n = 3048)	*p*-Value
Mean age at enrolled (SD), years	64.0 (9.4)	64.7 (10.5)	0.117
Age subgroup, n (%)			
50–59	311 (40.81)	1244 (40.81)	
60–69	234 (30.71)	936 (30.71)	
>70	217 (28.48)	868 (28.78)	1.000
Sex, n (%)			
Males	282 (37.01)	1128 (37.01)	
Females	480 (62.99)	1920 (62.99)	1.000
Charlson Comorbidity Index, n (%)			
0	22 (2.89)	88 (2.89)	
1–2	127 (16.67)	508 (16.67)	
3–4	222 (29.13)	888 (29.13)	
≥5	391 (51.31)	1564 (51.31)	1.000
Co-morbidity, n (%)			
Hyperlipidemia	518 (67.98)	1892 (62.07)	0.002
Hypertension	619 (81.23)	2033 (66.7)	<0.001
Heart failure	153 (20.08)	473 (15.52)	<0.001
Coronary artery disease	128 (16.80)	390 (12.80)	0.003
Chronic obstructive pulmonary disease	393 (51.57)	1246 (41.47)	0.002
Traumatic brain injury	189 (24.80)	470 (15.42)	<0.001
Depression	271 (35.56)	554 (18.18)	<0.001
Parkinson’s disease	68 (8.92)	111 (3.64)	<0.001
Stroke	362 (47.51)	377 (12.37)	<0.001
Medication, n (%)			
Anti-convulsant	653 (85.70)	578 (18.96)	<0.001
Anti-depressants	155 (20.34)	190 (6.23)	<0.001
Baclofen	526 (69.03)	1137 (37.30)	<0.001

IQR, interquartile range; SD, standard deviation.

**Table 2:** Characteristics of dementia events between trigeminal neuralgia (TN) patients and non-TN patients.

Characteristics	With Trigeminal Neuralgia (n = 762)	Without Trigeminal Neuralgia (n = 3048)	*p*-Value
Dementia event, n (%)	84 (11.02)	195 (6.40)	<0.001
Period of developing dementia median (IQR), years	3.7 (2.2–6.5)	11.4 (7.8–13.4)	<0.001
Mean age of dementia (SD), years	72.2 (8.4)	76.3 (10.3)	<0.001

**Table 3:** Risk of dementia events between trigeminal neuralgia (TN) patients and non-TN patients.

Variables	With Trigeminal Neuralgia			Without Trigeminal Neuralgia			Crude HR (95% CI)	Adjusted HR * (95% CI)
	Dementia Events	PYs	Rate	Dementia Events	PYs	Rate		
Overall	84	4384.18	19.16	195	44,822.76	4.35	7.94 (5.92–10.67)	4.47 (3.09–6.47)
Sex								
Males	30	1608.42	18.65	81	16,583.3	4.88	7.85 (4.82–12.79)	4.24 (2.29–7.83)
Females	54	2775.76	19.45	114	28,239.49	4.04	8.04 (5.55–11.65)	4.78 (3.00–7.61)
Age subgroup								
50–59	9	1776.96	5.06	14	18,586.11	0.75	12.85 (5.48–30.10)	6.07 (2.52–14.62)
60–69	18	1477.93	12.18	41	13,833.41	2.96	7.32 (4.14–12.96)	3.74 (2.02–6.92)
≥70	57	1129.23	50.48	140	12,403.24	11.29	8.58 (6.08–12.11)	4.57 (3.04–6.88)

Rate, the incidence rate per 1000 person-years; CI, confidence interval; HR, hazard ratio. * Model adjusted for age, sex, Charlson comorbidity index score, relevant comorbidities, and medications.

## Data Availability

All data generated or analyzed during this study are included in this published article.

## Supplementary Materials

The following supporting information can be downloaded at https://www.mdpi.com/article/10.3390/ijerph19106073/s1, Table S1: Significant predictors of dementia after trigeminal neuralgia diagnosis; Table S2: Joint effect of trigeminal neuralgia and comorbidities on dementia risk.
