# Supplementary material for: Trigeminal Neuralgia Is a Dementia Risk Factor: A Retrospective Cohort Study
Source: Int J Environ Res Public Health. 2022 May 17;19(10):6073. doi: 10.3390/ijerph19106073 (PMC9142042; doi:10.3390/ijerph19106073)
Supplement: Supplementary file 1 [file ijerph-19-06073-s001.zip › ijerph-1685089-supplementary.pdf]

Table S1. Significant predictors of dementia after trigeminal neuralgia diagnosis.

Variables	Adjusted HR*	95% CI	P-value
**Age**	2.89	(2.08-4.04)	<0.001
**Depression**	2.80	(1.77-4.44)	<0.001
**Parkinson's disease**	1.99	(1.23-3.21)	0.005
**Charlson Comorbidity Index score**	1.76	(1.13-2.75)	0.012
**Traumatic brain injury**	1.81	(1.15-2.86)	0.011
**Coronary artery disease**	1.70	(1.08-2.68)	0.022

HR, hazard ratio; 95% CI, 95% confidence interval. *Adjusted HR and 95% CI were estimated by stepwise Cox proportional hazards regression method; the model was adjusted for age, sex, Charlson Comorbidity Index score, relevant comorbidities, and medications.

Table S2. Joint effect of trigeminal neuralgia and comorbidities on dementia risk.

Variables		*n*	Dementia	Rate	Adjusted HR* (95% CI)
**Trigeminal neuralgia**	**Depression**				
No	No	2494	120	3.24	1.00 (Reference)
No	Yes	554	75	9.49	2.03 (1.49-2.74)[&]
Yes	No	491	29	10.13	3.68 (2.25-6.00)[&]
Yes	Yes	271	55	36.19	10.51 (6.78-16.28)[&]
**Trigeminal neuralgia**	**Parkinson's disease**				
No	No	2937	157	3.62	1.00 (Reference)
No	Yes	111	38	25.14	2.42 (1.67 -3.49)[&]
Yes	No	694	58	14.56	4.67 (3.11 -7.00)[&]
Yes	Yes	68	26	64.72	9.81 (5.89 -16.40)[&]
**Trigeminal neuralgia**	**Traumatic brain injury**				
No	No	2578	132	3.47	1.00 (Reference)
No	Yes	470	63	9.34	1.60 (1.18-2.18)[#]
Yes	No	573	52	15.59	4.45 (2.92-6.78)[&]
Yes	Yes	189	32	30.53	7.25 (4.47-11.87)[&]
**Trigeminal neuralgia**	**Coronary artery disease**				
No	No	2658	140	3.57	1.00 (Reference)
No	Yes	390	55	9.82	1.10 (0.79-1.54)
Yes	No	634	51	14.18	3.94 (2.57-6.02)[&]
Yes	Yes	128	33	41.85	6.14 (3.78-10.00)[&]

Rate, incidence rate per 1000 person-years; CI, confidence interval; HR, hazard ratio. * Model adjusted for age, sex, Charlson Comorbidity Index score, relevant comorbidities, and medications. [&] $p < 0.001$. [#] $p = 0.003$.
